# Assessing Physicians’ Recall Bias of Work Hours With a Mobile App: Interview and App-Recorded Data Comparison

**DOI:** 10.2196/26763

**Published:** 2021-12-24

**Authors:** Hsiao-Han Wang, Yu-Hsuan Lin

**Affiliations:** 1 Department of Psychiatry College of Medicine National Taiwan University Taipei Taiwan; 2 Department of Psychiatry National Taiwan University Hospital Taipei Taiwan; 3 Institute of Population Health Sciences National Health Research Institutes Miaoli County Taiwan; 4 Institute of Health Behaviors and Community Sciences College of Public Health National Taiwan University Taipei Taiwan

**Keywords:** smartphone, mobile app, work hours, recall bias, time perception, physicians, labor policy

## Abstract

**Background:**

Previous studies have shown inconsistencies in the accuracy of self-reported work hours. However, accurate documentation of work hours is fundamental for the formation of labor policies. Strict work-hour policies decrease medical errors, improve patient safety, and promote physicians’ well-being.

**Objective:**

The aim of this study was to estimate physicians’ recall bias of work hours with a mobile app, and to examine the association between the recall bias and physicians’ work hours.

**Methods:**

We quantified recall bias by calculating the differences between the app-recorded and self-reported work hours of the previous week and the penultimate week. We recruited 18 physicians to install the “Staff Hours” app, which automatically recorded GPS-defined work hours for 2 months, contributing 1068 person-days. We examined the association between work hours and two recall bias indicators: (1) the difference between self-reported and app-recorded work hours and (2) the percentage of days for which work hours were not precisely recalled during interviews.

**Results:**

App-recorded work hours highly correlated with self-reported counterparts (*r*=0.86-0.88, *P*<.001). Self-reported work hours were consistently significantly lower than app-recorded hours by –8.97 (SD 8.60) hours and –6.48 (SD 8.29) hours for the previous week and the penultimate week, respectively (both *P*<.001). The difference for the previous week was significantly correlated with work hours in the previous week (*r*=–0.410, *P*=.01), whereas the correlation of the difference with the hours in the penultimate week was not significant (*r*=–0.119, *P*=.48). The percentage of hours not recalled (38.6%) was significantly higher for the penultimate week (38.6%) than for the first week (16.0%), and the former was significantly correlated with work hours of the penultimate week (*r*=0.489, *P*=.002)

**Conclusions:**

Our study identified the existence of recall bias of work hours, the extent to which the recall was biased, and the influence of work hours on recall bias.

## Introduction

Excessive work hours adversely affect physicians’ alertness and performance [1], increase the number of medical errors, and jeopardize patient safety [2]. The crucial effects of work-hour policies on patient safety have been widely described since 2003 when the Accreditation Council for Graduate Medical Education (ACGME) adopted restrictions on physician work hours to 80 hours a week and no more than 24 consecutive hours [2-6]. Due to similar concerns, in 2019, the work-hour restrictions in Taiwan became stricter, from a limit of 88 hours per week adopted in 2013 to 80 hours per week and 28 hours of continuous work duty.

ACGME’s regulations shifted following evidence provided by trials and systematic reviews. In 2003, the ACGME allowed 24 hours of continuous work duty, which was reduced to 16 hours in 2011 and then reverted back to 24 hours in 2017 following results of a randomized controlled trial published in 2016 showing that more flexible duty-hour policies resulted in noninferior patient outcomes and physicians’ self-reported well-being when compared with restrictive policies [4]. In addition, a recent study showed that residents on 16-hour or less schedules made more serious medical errors than those working shifts spanning 24 hours or more [7].

The controversial effects of eliminating extended-duration work shifts for physicians on patient safety might result from the methodological limitations of the measurement of work hours. Previous studies of work policies focused disproportionately on consecutive work hours of night shifts rather than on total work hours within a time frame, such as work hours per week. These studies were mostly limited by using work-hour measurements yielded from self-reported or medical staff–recorded logs [7] and described the fluctuating, inconsecutive nature of physician work hours with consecutive work hours. Self-reported work hours is a widely used metric in most research [3,8,9], although it is continuously shown to be unsuitable for monitoring over longitudinal time periods. Moreover, the value of self-reported work hours might be reduced by biases, especially in the estimation of total work hours. In addition, programs’ compliance with ACGME regulations are usually based on medical residents’ self-reports, which might be prone to the residents’ biases [10]. Resident physicians, of various proportions, have admitted to not reporting duty hours accurately so as to appear compliant with regulations [6,9].

Currently, the widespread use and deep reach of smartphones in modern life enable the measurement of work hours in an affordable, reliable, and unobtrusive way. We developed an app, “Staff Hours,” to automatically calculate a user’s work hours via GPS background data [11]. Staff Hours is a region-restricted app, which could only be downloaded in Taiwan. Staff Hours records consecutive work hours in real time with accuracy, and enables comparisons of work hours among different hospitals, departments, and divisions with aggregate data collected from the Staff Hours database.

Using Staff Hours, we assessed physicians’ estimation of work hours and found a clear bias toward underestimation. The specific aims of this study were to (1) identify recall bias indicators, including the percentage of days that work hours were not precisely recalled during interviews (NR) and the difference between self-reported and app-recorded work hours (D); and (2) examine the correlation between these two recall bias indicators and app-recorded work hours. We hypothesized that the two recall bias indicators can effectively demonstrate how work hours influence recall bias differently during the previous week and the penultimate week before assessment.

## Methods

### GPS-Defined Work Hours Recorded by the App

**Figure 1:**
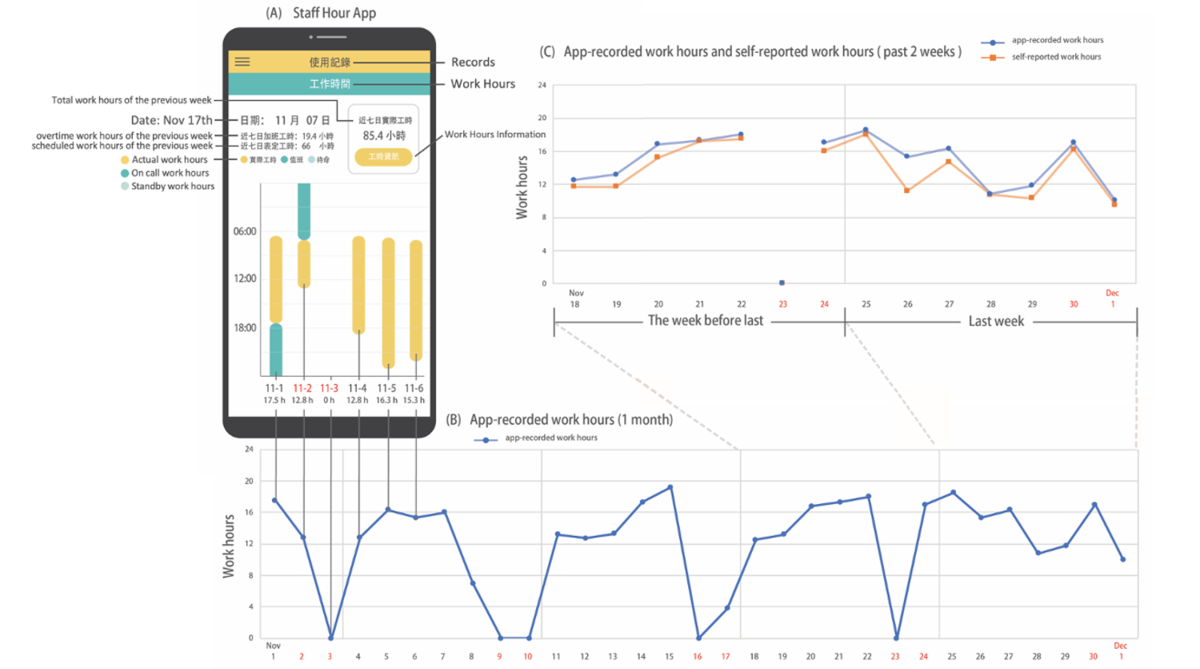
Screenshot of the Staff Hours app user interface, with app-recorded and self-reported work hours. (A) Weekly summary of daily work hours. The light gray bar represents regular work and the dark gray bar indicates on-call duty (night shift). (B) The Staff Hours app continuously collects GPS data without active data entry by the smartphone users and provides the daily work hours. (C) Recall bias was quantified by calculating the differences between the app-recorded (orange line) and self-reported (blue line) work hours of the last week (November 25 to December 1) and the penultimate week (November 18 to 24).

Staff Hours is a newly designed app that captures the work hours and patterns of medical staff in real time [11]. Participants installed the Staff Hours app onto their smartphones from Apple’s App Store or Android’s Google Play store. This app collects objective GPS location data continuously in the background and has a power-saving design. Using geofencing technology, the app automatically records the work hours one spends in the workplace. The sensitivity (94.6%) and specificity (93.9%) of app-recorded work hours were validated in our previous study [11]. The app is illustrated in Figure 1A. For example, the user’s regular work hours (6:30 AM to 5:30 PM) and on-call duty (5:30 PM to 12:00 AM) were 11 hours and 6.5 hours, respectively, on November 1; the sum of the work hours, 17.5 hours, was calculated by the app. The lower part of the screenshot reveals the total work hours of each day of the previous week. The upper part of the screenshot shows the regular work hours (66 hours) and overtime work hours (19.4 hours) on the left side, and the total work hours (85.2 hours) of the past 7 days at the top-right corner of the screen. App-generated data provide real-time work hours recorded with high temporal resolution (within 10 minutes). The app saves all recorded GPS data in a log file and uploads these data to the database every day. Figure 1B shows an example of recording the daily work hours from November 1 to December 1. This user typically had longer work hours during on-call duties (night shift), including November 1-2, 7-8, 21-22, and 24-25, as well as November 30 to December 1, on which days the scheduled works hours were 16 hours (8:00 AM to 12:00 AM). There were 10 holidays within this period of time, denoted with red figures on the x-axis. On five holidays (November 3, 9, 10, 16, 23), the user was free from on-call duty, and the work hours were 0. App-recorded work hours of the previous week (WH_APP-1_), the week before that (WH_APP-2_), and the previous month (WH_APP-M_, denoted by average work hours per week in 1 month), as well as the mean (SD) work hours of regular work days within a month, were obtained from app-record data. The app-recorded work hours were used as a putative gold standard to validate self-reported work hours.

### Participants

A total of 18 medical doctors (11 men; median age 29 years, range 24-53 years) were recruited by randomly selecting resident physicians working at hospitals in Taipei from August 2019 to December 2019. Approval was obtained from the ethics committee in Taiwan (approval number: EC1070107-E). The inclusion criteria were resident physicians who could install the Staff Hours app on their mobile phones. There were no strict exclusion criteria. Each participant installed the app and ran it for at least 2 months. The study duration was 61 days, contributing 1098 person-days. Some of the participants failed to record their work hours for technical reasons. When this happened, their data were considered missing data. There were 30 missing days. Hence, the final data included 1098–30=1068 person-days. The participants received identical structured interviews at the first and second month after installation of the Staff Hours app. The majority (16/18, 89%) of participants were resident physicians undertaking training programs in specialized fields, including surgery, internal medicine, pediatrics, obstetrics and gynecology, emergency medicine, occupational and environmental medicine, and psychiatry. All participants were given detailed descriptions regarding the study, and individual informed consent was obtained in written form. All clinical investigations were conducted according to the principles expressed in the Declaration of Helsinki. The study was approved by the Institutional Review Board of the National Health Research Institutes.

### Self-Reported Work Hours

The two authors, who are psychiatrists experienced in physicians’ work-hour patterns and structured interviews, conducted structured interviews at the first and second month after the app installation blinded to the app-recorded work hours. An identical structured interview was repeated 1 month after the first interview. The interrater reliability of WH_self-1_, WH_self-2_, NR_1_, and NR_2_ between the two interviewers was 1.00 in all cases based on Pearson correlation analysis. The lengths of both interviews were documented. The interviews simulated investigations conducted by the Taiwan Ministry of Labor. The definitions of work hours and nonwork hours (eg, continuing medical education) were in line with the standard policies in hospitals in Taiwan [12]. The first interview assessed recalled work hours of the past month. In this case, the interviewers recorded the average time of arriving and leaving work on regular work days over the past month, as well as how many work days and on-call days, respectively, there had been. The total work hours of the previous month were calculated by the obtained report. The second interview assessed the recalled work hours of the previous week and the penultimate week. In this case, the interviewers recorded the specific work hours of each day (from Monday to Sunday) during the previous week and the penultimate week, as demonstrated in Figure 1C. Recall bias was quantified by calculating the differences between the app-recorded (orange line) and self-reported (blue line) work hours of the last week (November 25 to December 1) and the penultimate week (November 18 to 24). In the example shown in Figure 1C, self-reported work hours were mostly lower than the app-recorded counterparts across time, by an average of 1.19 hours and 1.01 hours every day of the last week and the penultimate week, respectively.

In addition, the percentage of days that participants were unable to recall work hours precisely (NR) was recorded. If the participants reported they were unable to recall the work hours of a particular day, the interviewers provided cues by offering them their average time of arriving and leaving work in the last month according to the participant’s report earlier during the interview. Self-reported work hours of the previous week (WH_self-1_) and the penultimate week (WH_self-2_) were calculated by summing the reported work hours of each day.

### Recall Bias Indicators

We used two groups of indicators to quantify recall bias. The first group included the differences between self-reported and app-recorded work hours of the previous week (D_1_), penultimate week (D_2_), and previous month (D_M_), in which D is defined as WH_self_–WH_app_. The second group included the percentage of days that participants were unable to precisely recall their work hours of the previous week (NR_1_) and the penultimate week (NR_2_) during interviews.

### Statistical Analysis

We compared the self-reported work hours with their app-recorded counterparts with a paired *t* test, and examined their correlation with Pearson correlation coefficients. We compared the percentage of days that participants were unable to recall their work hours precisely for the previous week (NR_1_) and the penultimate week (NR_2_) with a paired *t* test. We also used Pearson correlation coefficients to examine the associations between work hours and the recall bias indicators D_1_, D_2_, NR_1_, and NR_2_. In addition, we examined the test-retest reliability of the recall bias indicators by intraclass correlation coefficients between the first month and the second month; *P*<.05 was considered to indicate statistical significance. Data arrangement and statistical analysis were performed using IBM SPSS Statistics 25.

## Results

An average of 9.7 (SD 3.2) minutes was required to complete a participant’s interview every month. The percentage of days that participants were unable to precisely recall their work hours of the penultimate week (NR_2_; mean 38.6%, SD 33.9%) was significantly higher than that of the previous week (NR_1_; mean 16.0%, SD 23.7%) by 18.5% (*P*=.004). The standard deviation of day-to-day work hours was 3.8 hours.

Both WH_app-1_ and WH_app-2_ presented a normal distribution according to the Kolmogorov-Smirnov test (*P*=.20 for both) and Shapiro-Wilk tests (WH_app-1_ *P*=.26, WH_app-2_ *P*=.77). These self-reported work hours were highly correlated to their app-recorded counterparts for the previous week (*r*=–0.87, *P*<.001), penultimate week (*r*= 0.88, *P*<.001), and previous month (*r*=0.86, *P*<.001). Table 1 shows that the self-reported hours were significantly lower than their app-recorded counterparts, with the greatest average differences for the previous week (D_1_), followed by the penultimate week (D_2_), and the smallest difference found for the previous month (D_M_) (all *P*<.001).

**Table 1:** Comparison of self-reported and app-recorded work hours of the previous week, the penultimate week, and previous month.

Time period recorded	Self-reported work hours	App-recorded work hours	Difference, mean (SD)	*P* value
Previous week	57.30	65.63	–8.33 (8.95)	<.001
Penultimate week	58.57	65.65	–7.08 (8.74)	<.001
Previous month (weekly)	60.26	66.94	–6.68 (8.27)	<.001

Table 2 shows the correlation coefficients between the recall bias indicators (D_1_, D_2_, NR_1_, NR_2_) and the app-recorded work hours (WH_app-1_ and WH_app-2_). WH_app-1_ was significantly negatively correlated to D_1_, meaning that the longer WH_app-1_, the more negative the difference between WH_self-1_ and WH_app-1_, representing more underestimation of self-reported work hours. WH_app-1_ was not significantly correlated to NR_1_. By contrast, WH_app-2_ was significantly correlated to NR_2_, meaning that the longer WH_app-2_, the higher percentage of days that work hours were not precisely recalled during interviews. WH_app-2_ was not correlated to D_2_ and WH _app-m_ was not significantly correlated to D_M_.

**Table 2:** Pearson correlation coefficients (r) of app-recorded work hours and recall bias indicators.

Recall bias indicator	WH_app-1_^a^		WH_app-2_^b^	
	*r*	*P* value	*r*	*P* value
Difference between self-reported and app-recorded work hours	–0.41	.01	–0.12	.48
Percentage of days that participants were unable to recall their work hours precisely	0.08	.37	–0.49	.002

^a^app-recorded work hours of the previous week.

^b^app-recorded work hours of the penultimate week.

## Discussion

### Principal Findings

This study demonstrated the underestimation of 11.5%-12.0% (D_1_/WH_app-1_ and D_2_/WH_app-2_) work hours and the association between this recall bias and excessive work hours, with the novel app “Staff Hours” recording GPS-defined work hours. We recruited a total of 18 medical doctors (11 men; median age 29 years, range 24-53 years) as participants. As methodological strengths, this study helps to advance the field of work-hours estimation by recording work hours in efficient, precise ways and with higher temporal resolution. The app automatically recorded work hours in real time, and an average of 9.7 minutes was required to complete a participant’s interview every month. We were able to collect self-reported data with structured interviews twice per participant, using psychiatrists as interviewers. An earlier study conducted in the United States in 2004 including 45 female flight attendants as participants examined work hours per month [13]. Another study conducted in Japan in 2016 included 164 male employees as participants to gather work-hour information with questionnaires [14]. Studies using self-reported questionnaires typically processed work-hour details as ordinal variables [14], such as by recording the work hours roughly (eg, 45 to <60 hours per week, 60 to <80 hours per week, and >80 hours per week) rather than as continuous variables (eg, 67 hours per month), although the latter has higher precision than using ordinal variables. In addition, the app recorded data with higher temporal resolution than obtained from self-reports. This study included 18 participants and ran over 61 work days, contributing 1068 pairs of app-recorded and self-reported data after subtracting missing data. On the basis of the high temporal resolution of the data, the standard deviation of the average day-to-day work hours was 3.8 hours, highlighting the fluctuating nature of physicians’ work hours.

Our result that app-recorded work hours strongly correlated with their self-reported counterparts (*r*=0.86-0.88, *P*<.001) was consistent with previous studies [2,14]. One previous study [14] also demonstrated a high correlation between self-reported and actual work hours. Our study further extends this previous research by describing the extent of underestimation; the weekly underestimation was 6.48 to 8.97 work hours per week, which represents approximately 1.30-1.79 hours of underestimation every day considering a 5-day work week (6.48/5=1.30; 7.05/5=1.79). Differences of temporal resolution between the app-recorded work hours and participants’ recall were noted, which may contribute to systematic biased recall. When participants were unable to recall the work hours of a particular day precisely, they tended to report rounded-up hours, typically to the nearest half hour (eg, reporting as having arrived at work at 6:30 AM or 7:00 AM), as well as reporting regular work hours, which were often lower than their actual work hours. The app scanned at 10-minute intervals to determine the GPS coordinates and therefore the initiation and cessation of recording work hours. Our results showed that all participants reported starting at the scheduled work time, despite actually having arrived at the workplace a few minutes earlier than their work hours according to GPS coordinates.

Previous studies [9,10] showed that when duty hour regulations were enforced, resident physicians were faced with a dilemma between violating the duty hour regulations and maintaining patient care quality. When facing such a dilemma, an option that resident physicians chose was working overtime while still reporting the duty hours within the limitations of regulations. It was estimated that up to 60% of surgical residents reported their work hours untruthfully when filling out questionnaires. The reasons resident physicians underreported their work hours might have included pressure from the system (eg, receiving punishment due to violations of regulations) and pressure from peers (eg, being viewed as “incompetent” when leaving work on time).

Besides differences in temporal resolution between app-recorded and self-reported work hours, distorted time perception resulting from long work hours may also contribute to recall bias, as demonstrated by the two recall bias indicators used in this study. Physicians whose work hours were longer during the previous week demonstrated a tendency toward more underestimation during recall (*r*=–0.410, *P*=.01), whereas participants whose work hours were longer in the penultimate week showed a diminished tendency to underestimate their work hours during recall (*r*=–0.119, *P*=.48). In addition to previous studies that simply showed the extent to which recall of work hours was impaired [2,14], our study recorded the percentage of work days that participants were unable to recall their work hours. Participants whose work hours were longer during the penultimate week showed a lower rate of recalling their work hours precisely when compared with those who worked shorter hours (*r*=0.489, *P*=.002). As a result of a higher NR, the interviewers provided cues more frequently when participants recalled their work hours of the penultimate week, thus allowing for attenuation of underestimation.

Excessive work hours may distort the time perception and memory of the extent of work hours. Our previous studies performed in 2012 involving 74 medical interns showed that physicians in Taiwan worked an average of 86.7 hours per week with up to 33.5 consecutive working hours per duty shift, and that they sometimes developed hypervigilant perceptions, phantom vibration, and ringing-ear syndrome in the absence of an external stimulus [1,15]. Excessive work hours also resulted in reduced cardiac sympathetic modulation [1,16], disrupted sleep stability [17], and increased anxiety and depression symptoms [18-20]. These psychological and physiological impacts of excessive work hours could alter time memory and time perception (ie, the ability to recall work hours), thereby increasing recall bias [21,22].

### Limitations

This study has several methodological limitations that should be considered when interpreting the results. First, if the participants reported that they were unable to recall the work hours of a particular day, the interviewers provided cues by offering them their average time of arriving and leaving work in the last month. For some participants who were more unable to recall their work hours (ie, those with a larger NR), their D values were calculated based more on cues provided by the interviewers. By contrast, NR was not affected by any cues provided by the interviewers. Therefore, NR might be a better self-defined recall bias indicator compared with D. Second, the participants were not asked to recall any work hours earlier than the penultimate week, which thus limited the studied timeframe of recall bias in this study. However, the percentage of days that participants were unable to recall work hours of the penultimate week was 38.6%. It was conceivable that reports of work hours earlier than 2 weeks prior may result in greater recall bias. Third, the sample size of this study was relatively small, which limits the generalization of the findings as well as the detection of other recall bias indicators. However, we were able to ensure participant diversity, and the 2-month length of app installation guaranteed adequate data input for analysis. Future research should investigate how a larger sample size would identify more factors associated with recall bias indicators. Future studies are also warranted to assess the underlying mechanisms of time perception and recall bias of work hours.

### Conclusion

This pilot study identified the existence of recall bias of work hours, the extent to which the recall was biased (6.48-8.97 work hours per week), and the influence of work hours on recall bias. We were able to demonstrate that long work hours in the previous and the penultimate week influenced recall bias in different ways. Working overtime has long been part of the culture among Taiwanese physicians [1,15,17,19,20,23]. However, the extent to which medical staff overwork has never been systemically investigated, and hence the work-hour regulations of physicians were formed without the basis of solid work-hour studies. This study has clear public health relevance by confirming that this time-saving, easily accessible app can provide solid, precise reports of actual work hours, aid work policy formation for physicians, and promote the care quality of patients, as well as the well-being of physicians in Taiwan.

## Abbreviations

ACGME: Accreditation Council for Graduate Medical Education
D: difference between self-reported and app-recorded work hours
NR: percentage of days that work hours were not precisely recalled during interviews
WH: work hours
